# Predictors and outcomes of patient safety culture in hospitals

**DOI:** 10.1186/1472-6963-11-45

**Published:** 2011-02-24

**Authors:** Fadi El-Jardali, Hani Dimassi, Diana Jamal, Maha Jaafar, Nour Hemadeh

**Affiliations:** 1 Department of Health Management and Policy, Faculty of Health Sciences, American University of Beirut, Beirut, Lebanon; 2 School of Pharmacy, Lebanese American University Beirut, Lebanon; 3 Bachelors Program on Political Sciences and Public Administration, Faculty of Arts and Sciences, American University of Beirut, Beirut, Lebanon

## Abstract

**Background:**

Developing a patient safety culture was one of the recommendations made by the Institute of Medicine to assist hospitals in improving patient safety. In recent years, a multitude of evidence, mostly originating from developed countries, has been published on patient safety culture. One of the first efforts to assess the culture of safety in the Eastern Mediterranean Region was by El-Jardali et al. (2010) in Lebanon. The study entitled "The Current State of Patient Safety Culture: a study at baseline" assessed the culture of safety in Lebanese hospitals. Based on study findings, the objective of this paper is to explore the association between patient safety culture predictors and outcomes, taking into consideration respondent and hospital characteristics. In addition, it will examine the correlation between patient safety culture composites.

**Methods:**

Sixty-eight hospitals and 6,807 respondents participated in the study. The study which adopted a cross sectional research design utilized an Arabic-translated version of the Hospital Survey on Patient Safety Culture (HSOPSC). The HSOPSC measures 12 patient safety composites. Two of the composites, in addition to a patient safety grade and the number of events reported, represented the four outcome variables. Bivariate and mixed model regression analyses were used to examine the association between the patient safety culture predictors and outcomes.

**Results:**

Significant correlations were observed among all patient safety culture composites but with differences in the strength of the correlation. Generalized Estimating Equations for the patient safety composite scores and respondent and hospital characteristics against the patient safety grade and the number of events reported revealed significant correlations. Significant correlations were also observed by linear mixed models of the same variables against the frequency of events reported and the overall perception of safety.

**Conclusion:**

Event reporting, communication, patient safety leadership and management, staffing, and accreditation were identified as major patient safety culture predictors. Investing in practices that tackle these issues and prioritizing patient safety is essential in Lebanese hospitals in order to improve patient safety. In addition, further research is needed to understand the association between patient safety culture and clinical outcomes.

## Background

Developing a patient safety culture was one of the recommendations made by the Institute of Medicine to assist hospitals in improving patient safety [1,2]. Assessing the organization's existing safety culture is the first stage of developing a safety culture [3]. Patient safety culture assessments, required by international accreditation organizations, allow healthcare organizations to obtain a clear view of the patient safety aspects requiring urgent attention, identify the strengths and weaknesses of their safety culture [4], help care giving units identify their existing patient safety problems [5], and benchmark their scores with other hospitals [6].

According to literature, the major predictors of a positive patient safety culture in healthcare organizations specifically hospitals include communication founded on mutual trust, good information flow, shared perception of the importance of safety, organizational learning, commitment from management and leadership, and the presence of a non-punitive approach to incident and error reporting [7]. Patient safety culture outcomes include the staff members' perception of safety, the willingness of staff members to report events, the number of events reported, and an overall patient safety grade given by staff members to their units [8].

A multitude of evidence has been published in the area of patient safety culture in recent years. Some of the available evidence tackles patient safety culture issues that require attention, factors affecting incident reporting by hospital staff, the role of workplace environment in shaping safety, and steps that can be followed to improve safety.

Despite the wealth of evidence on patient safety culture, limited evidence still exists about the linkage between predictors and outcomes of patient safety culture especially in countries of the Eastern Mediterranean Region. One of the first efforts to assess the culture of safety in hospitals in the region was conducted in Lebanon by El-Jardali et al. [9].

### Lebanese Context

The study by El-Jardali et al. (2010) entitled "The Current state of Patient Safety Culture in Lebanese Hospitals: A study at Baseline" [9] utilized an Arabic translated version of the *Hospital Survey on Patient Safety Culture *(HSOPSC) [8]. It aimed at identifying the most critical issues related to patient safety culture and potential strategies to implement the patient safety accreditation standards in the light of a newly added chapter to the Lebanese handbook of hospital accreditation [10].

The HSOPSC measures 12 patient safety culture composites representing several patient safety culture predictors (See Box 1). The HSOPSC also requires respondents to give their work area/unit a patient safety grade and to answer a question on the number of events reported in the past 12 months [8].

Calculating the percentage of positive responses for each composite revealed that the composites with the highest positive ratings were teamwork within units, hospital management support for patient safety, and organizational learning and continuous improvement. However, composites with the lowest ratings were teamwork across hospital units, hospital handoffs and transitions, staffing, and non-punitive response to error [9]. Approximately 60% of respondents reported not completing any event reports in the past 12 months and over 70% gave their units an "Excellent/Very Good" patient safety grade. Bivariate and multivariate analyses revealed significant differences across hospitals of different size and accreditation status [9].

Study findings outlined above represent the first component of data analysis for that stage [9]. Findings provided evidence that communication across units, staffing, event reporting, and the culture of response to error were major patient safety culture issues [9]. This paper, though, will further explore the association between patient safety culture predictors and outcomes, taking into consideration respondent and hospital characteristics. In addition, it will examine the correlation between the patient safety culture composites. Thus, the objective of this paper is to address the afore-mentioned objectives using bivariate as well as multivariate analyses.

## Methods

### Study Design, Setting, and Sample

The study adopted a cross sectional research design utilizing a customized version of the HSOPSC. A pilot testing phase preceded data collection in order to ensure the validity and reliability of the Arabic translated version of the questionnaire. Of the 126 hospitals registered in the Lebanese Syndicate of Private Hospitals that were contacted and asked to participate, 68 consented [9]. The survey targeted hospital employees including physicians, nurses, clinical and non-clinical staff, pharmacy and laboratory staff, dietary and radiology staff, supervisors, and hospital managers. The questionnaire was distributed to 12,250 hospital employees and 6807 were returned yielding an overall response rate of 55.56%. Additional details on the study methodology can be found in El-Jardali et al. [9].

### Survey Measures and Outcome Variables

The HSOPSC is composed of 42 items that measure 12 composites of patient safety culture (See Box1, Additional file1 ). Items were scored using a five-point scale reflecting the agreement rate on a five-point frequency scale. The percentage of positive responses for each item was calculated; negatively worded items were reversed when computing percent positive response. Composite level scores were computed by summation of the items within the composite scales and dividing by the number of items with non-missing values [9].

Two of the composites (frequency of events reported and overall perception of safety) are two of the four patient safety culture outcome variables [8]. The remaining two outcome variables are the patient safety grade and the number of events reported [8].

### Bivariate Analysis

Bivariate analyses were used to examine the associations between patient safety culture composites and differences across hospitals of different size and accreditation status.

Pearson correlations were used to examine the association between the patient safety culture composites. ANOVA f-test with multiple comparison corrected using the Bonferroni method was used to examine the association between the two outcome variables (patient safety grade and the number of events reported) with the remaining patient safety culture composites.

Student T-Test and ANOVA f-test with multiple comparison corrected using the Bonferroni method were then used to examine how trends in the outcome variables (frequency of events reported and overall perception of safety) differ across hospital and respondent characteristics. Finally, cross tables were constructed and chi-square tests were used to assess how trends in the outcome variables (patient safety grade and the number of events reported) differed across respondent and hospital characteristics.

### Multivariate Regression Analysis

The four outcome variables were regressed against the 10 composite scores, respondent's position in the hospital, accreditation status, and hospital size. Since the data was clustered by hospital, we used appropriate statistical techniques to control for this effect. Four regression models were constructed, two adopted Generalized Estimating Equations (the two categorical outcome variables: number of events reported and patient safety grade) and the other two models followed a linear mixed regression model (the two composites for frequency of events reported and overall perception of safety).

## Results

### Comparison of Means for the Frequency of Events Reported and the Overall Perception of Safety across Respondent and Hospital Characteristics

Significant differences were observed between units and positions when comparing results across respondent and hospital characteristics. For both the frequency of events reported and the overall perception of safety, significantly higher means were observed for diagnostics (mean = 3.97 ± 1.01; mean = 3.96 ± 0.68) as compared to surgical (mean = 3.78 ± 1.05; mean = 3.82 ± 0.67) and medical units (mean = 3.93 ± 0.99; mean = 3.83 ± 0.68) (See Table 1).

**Table 1:** Comparison of means for two outcome composite scores across hospital and respondent characteristics *(identical letters represent significance between indicated groups)*

	Frequency of Events Reported			Overall Perception of Safety		
	Mean (SD)		P-Value	Mean (SD)		P-Value
**Unit**						
Many different hospital units/no specific unit	3.89 (0.96)	a, f	***<0.001***	3.72 (0.66)	a, d, e, f	***<0.001***
Administration	3.78 (1.16)	b, f		3.72 (0.75)	b, e,	
Medical	3.93 (0.99)	c, d		3.83 (0.68)	a, c, e	
Surgical	3.78 (1.05)	c, d, e, f		3.82 (0.67)	a, d, e,	
Diagnostics	3.97 (1.01)	b, d, e		3.96 (0.68)	a, b, c, e	
Other	4.06 (0.98)	a, b, d, f		3.91 (0.64)	a, b, f	
N	5707			5201		

**Position**						
Nurse	3.89 (1.00)	a, e, f	***<0.001***	3.80 (0.66)	a, c	***<0.001***
Physician	3.78 (0.92)	b, f		3.69 (0.75)	b, c, d	
Pharmacist	3.87 (1.20)			3.83 (0.90)		
Other health professions	3.95 (0.93)	c, e,		3.90 (0.77)		
Unit assistant/clerk/secretary/Technician	3.92 (1.05)	d, e,		3.92 (0.68)	a, b	
Administration	3.92 (1.06)			3.75 (0.85)		
Quality and Safety	3.49 (1.06)	a, c, d, e		3.86 (0.63)		
Other	4.04 (0.99)	a, b, e, f		3.90 (0.71)	b, d	
N	5925			5407		

**Experience at hospital**						
Less than 1 year	3.78 (1.08)	a, b, c, d	***0.001***	3.81 (0.72)		***0.003***
1 to 5 years	3.88 (1.00)			3.82 (0.67)		
6 to 10 years	3.94 (1.00)	a, b		3.83 (0.67)		
11 to 15 years	3.98 (0.98)	a, c		3.88 (0.65)	a	
16 to 20 years	3.91 (1.04)			3.82 (0.69)		
21 years or more	4.00 (1.02)	a, d		3.74 (0.79)	a	
N	6132			5573		

**Hospital Size (El-Jardali et al., 2010)**						
Small (<100 beds)	3.95 (1.00)	a, b, c	***0.001***	3.84 (0.67)	a	***0.012***
Medium (100-199 beds)	3.87 (1.03)	a, b		3.80 (0.69)		
Large (>=200 beds)	3.81 (1.08)	a, c		3.76 (0.71)	a	
N	6307			5742		

**Interaction with patients**						
Yes	3.90 (1.00)		0.517	3.82 (0.68)		0.092
No	3.93 (1.05)			3.87 (0.73)		
N	5707			5201		

**Accreditation Status (El-Jardali et al., 2010)**						
Yes	3.91 (1.03)		***0.047***	3.84 (0.69)		***<0.001***
No	3.84 (1.03)			3.71 (0.68)		
N	6307			5742		

Significantly higher means were observed for nurses (mean = 3.89 ± 1.00; mean = 3.80 ± 0.66) than physicians (mean = 3.78 ± 0.92; mean = 3.69 ± 0.75) on the frequency of events reported and the overall perception of safety respectively (See Table 1).

For frequency of events reported, higher means (and thus more events) were observed for increasing years of experience whereas the opposite trend prevailed for overall perception of safety (See Table 1). Small hospitals (mean = 3.95 ± 1.00) had a significantly higher frequency of events reported in comparison to medium (mean = 3.87 ± 1.03) and large hospitals (mean = 3.81 ± 1.08). Small hospitals (mean = 3.84 ± 0.67) also had a better overall perception of safety than large hospitals (mean = 3.76 ± 0.71). Accredited hospitals (mean = 3.91 ± 1.03) had a significantly higher frequency of events reported than non-accredited hospitals (mean = 3.84 ± 1.03). Accredited hospitals (mean = 3.84 ± 0.69) also had a better overall perception of safety than non-accredited hospitals (mean = 3.71 ± 0.68) (See Table 1).

### Correlations between Patient Safety Culture Composites

While significant correlations were observed among all patient safety culture composites, differences in the strength of the correlation were observed (See Table 2). The composite measuring staffing showed the weakest correlation with the outcome on frequency of event reporting (Pearson r = 0.107). The composite measuring feedback and communication about errors had the strongest correlation with this outcome variable (Pearson r = 0.378) (See Table 2).

**Table 2:** Correlation between patient safety culture composites

	Frequency of Events Reported		Overall Perception of Safety	
	Pearson r	N	Pearson r	N
Supervisor/manager expectations and actions promoting safety	.206*	5878	.371*	5467
Organizational Learning-Continuous Improvement	.221*	5884	.303*	5542
Teamwork Within Hospital Units	.195*	6008	.308*	5531
Communication Openness	.267*	6017	.276*	5539
Feedback and Communication About Errors	.378*	6056	.288*	5555
Non-punitive Response to Error	.116*	5975	.179*	5560
Staffing	.107*	5168	.310*	4959
Hospital Management Support for Patient Safety	.243*	6160	.358*	5614
Hospital Handoffs and Transitions	.159*	5139	.355*	4832
Teamwork Across Hospital Units	.250*	5988	.297*	5498

*Correlation is significant at the 0.01 level (2-tailed).

The composite with the weakest correlation with the outcome variable measuring perceptions of patient safety was non-punitive response to error (Pearson r = 0.179) while that with the strongest correlation was the composite measuring supervisor/manager expectations and actions promoting safety (Pearson r = 0.371) (See Table 2).

### Comparison of Means between Outcome Variables and Patient Safety Composites

Significantly different means were reported for patient safety grade with all patient safety composite scores. Respondents who gave "Excellent/Very Good" patient safety grades had the highest mean scores for patient safety composites (See Table 3). The number of events reported was significantly associated with the composites measuring communication openness, feedback and communication about errors, non-punitive response to error, hospital handoffs and transitions, and teamwork across hospital units. The highest means were observed when reporting more than 5 events for the composites on communication openness, feedback and communication about errors, and non-punitive response to error. The opposite was observed for the composites measuring hospital handoffs and transitions and teamwork across hospital units where the highest means were observed when no events were reported (see Table 3).

**Table 3:** Comparison of means between patient safety grade and number of events reported with patient safety culture composite scores

	Patient Safety Grade				Number of Events Reported			
		Poor or Failing	Acceptable	Excellent/Very Good		No event reports	1 to 5 event reports	>5 events reported
	**Sig**.	Mean (SD)	Mean (SD)	Mean (SD)	**Sig**.	Mean (SD)	Mean (SD)	Mean (SD)
Supervisor/manager expectations and actions promoting safety	a,b,c	3.00 (0.84)	3.39 (0.69)	3.82 (0.62)		3.71 (0.67)	3.70 (0.67)	3.72 (0.72)
Organizational Learning-Continuous Improvement	a,b,c	3.26 (0.93)	3.72 (0.63)	4.04 (0.56)	c	3.93 (0.62)	3.96 (0.58)	4.02 (0.62)
Teamwork Within Hospital Units	a,b,c	3.37 (0.88)	3.75 (0.66)	4.14 (0.57)		4.02 (0.65)	4.04 (0.59)	4.04 (0.68)
Communication Openness	a,b,c	2.88 (0.92)	3.33 (0.86)	3.76 (0.80)	b,c	3.62 (0.87)	3.65 (0.81)	3.77 (0.87)
Feedback and Communication About Errors	a,b,c	2.88 (1.09)	3.51 (0.87)	4.12 (0.76)	a,b,c	3.90 (0.88)	3.97 (0.81)	4.08 (0.81)
Non-punitive Response to Error	b,c	2.42 (0.91)	2.46 (0.76)	2.66 (0.79)	b,c	2.58 (0.78)	2.62 (0.78)	2.74 (0.89)
Staffing	a,b,c	2.41 (0.80)	2.65 (0.68)	2.97 (0.75)		2.89 (0.77)	2.91 (0.73)	2.89 (0.76)
Hospital Management Support for Patient Safety	a,b,c	3.04 (0.97)	3.62 (0.71)	4.14 (0.65)		4.00 (0.72)	4.00 (0.71)	3.97 (0.83)
Hospital Handoffs and Transitions	a,b,c	2.80 (0.76)	3.03 (0.76)	3.49 (0.76)	b,c	3.40 (0.80)	3.34 (0.76)	3.19 (0.82)
Teamwork Across Hospital Units	a,b,c	2.64 (0.69)	3.09 (0.67)	3.56 (0.68)	b	3.44 (0.73)	3.42 (0.68)	3.34 (0.77)

Patient Safety Grade

a. Significant difference between "Poor or Failing" and "Acceptable"

b. Significant difference between "Poor or Failing" and "Excellent/Very Good"

c. Significant difference between "Acceptable" and "Excellent/Very Good"

Number of Events Reported

a. Significant difference between "No events reported" and "1 to 5 events reported"

b. Significant difference between "No events reported" and "> 5 events reported"

c. Significant difference between "1 to 5 events reported" and "> 5 events reported"

### Distribution of the Patient Safety Grade and the Number of Events Reported across Respondent and Hospital Characteristics

#### Patient Safety Grade

The highest percentage of respondents reporting "Excellent/Very Good" patient safety grades were those working in diagnostics (83.6%). The highest percentage of respondents reporting a "Poor/Failing" patient safety grade (4.2%) worked across many different hospital units. Although the majority of respondents gave their units an "Excellent/Very Good" patient safety grade, physicians (63.3%) and administrators (63.3%) were the least to report an "Excellent/Very Good" patient safety grade (See Table 4).

**Table 4:** Distribution of two outcome variables across hospital and respondent characteristics

	Patient Safety Grade							Number of Events Reported						
	Excellent or Very Good		Acceptable		Poor or Failing			No event reports		1 to 5 event reports		>5 events reported		
	N	%	N	%	N	%	P-Value	N	%	N	%	N	%	P-Value
**Work area/unit where respondents spend most of their work time**														
Many different hospital units/no specific unit	458	64.2%	225	31.6%	30	4.2%	***<0.001***	323	49.9%	266	41.1%	58	9.0%	***<0.001***
Administration	353	68.8%	142	27.7%	18	3.5%		311	63.5%	116	23.7%	63	12.9%	
Medical	1181	72.5%	414	25.4%	35	2.1%		831	57.6%	525	36.4%	86	6.0%	
Surgical	1049	72.3%	359	24.7%	43	3.0%		769	59.2%	457	35.2%	74	5.7%	
Diagnostics	602	83.6%	112	15.6%	6	0.8%		390	60.4%	202	31.3%	54	8.4%	
Other	480	81.8%	99	16.9%	8	1.4%		323	60.8%	156	29.4%	52	9.8%	

**Position at the hospital**														
Nurse	2581	71.3%	941	26.0%	99	2.7%	***<0.001***	1845	57.2%	1190	36.9%	191	5.9%	***<0.001***
Physician	148	63.3%	80	34.2%	6	2.6%		117	57.1%	71	34.6%	17	8.3%	
Pharmacist	53	77.9%	15	22.1%	0	0.0%		23	37.7%	22	36.1%	16	26.2%	
Other health professions	83	73.5%	25	22.1%	5	4.4%		61	55.5%	44	40.0%	5	4.5%	
Unit assistant/clerk/secretary/Technician	676	79.6%	162	19.1%	11	1.3%		521	68.4%	195	25.6%	46	6.0%	
Administration	107	63.3%	53	31.4%	9	5.3%		81	48.5%	59	35.3%	27	16.2%	
Quality and Safety	72	67.9%	30	28.3%	4	3.8%		41	41.8%	28	28.6%	29	29.6%	
Other	538	81.6%	115	17.5%	6	0.9%		374	61.0%	187	30.5%	52	8.5%	

**Years of experience at hospital**														
Less than 1 year	556	70.8%	203	25.9%	26	3.3%	***0.001***	476	68.7%	180	26.0%	37	5.3%	***<0.001***
1 to 5 years	1734	72.4%	586	24.5%	72	3.0%		1229	56.3%	788	36.1%	165	7.6%	
6 to 10 years	882	74.2%	289	24.3%	18	1.5%		599	55.5%	386	35.8%	94	8.7%	
11 to 15 years	625	78.2%	160	20.0%	14	1.8%		414	58.2%	246	34.6%	51	7.2%	
16 to 20 years	277	73.5%	96	25.5%	4	1.1%		197	60.6%	107	32.9%	21	6.5%	
21 years or more	343	73.1%	120	25.6%	6	1.3%		244	58.9%	136	32.9%	34	8.2%	

**Hospital Size (El-Jardali et al., 2010)**														
Small (<100 beds)	2214	73.1%	748	24.7%	66	2.2%	0.563	1600	58.2%	948	34.5%	199	7.2%	0.179
Medium (100-199 beds)	1751	74.0%	553	23.4%	61	2.6%		1235	59.1%	684	32.7%	172	8.2%	
Large (>=200 beds)	550	72.5%	187	24.6%	22	2.9%		389	58.2%	240	35.9%	39	5.8%	

**Interaction with patients**														
Yes	3562	73.1%	1196	24.5%	119	2.4%	0.153	2486	56.9%	1574	36.0%	310	7.1%	***<0.001***
No	805	75.6%	239	22.4%	21	2.0%		648	65.1%	266	26.7%	81	8.1%	

**Hospital Accreditation Status (El-Jardali et al., 2010)**														
Yes	3871	74.2%	1224	23.5%	121	2.3%	***<0.001***	2687	57.5%	1621	34.7%	361	7.7%	***0.001***
No	644	68.8%	264	28.2%	28	3.0%		537	64.2%	251	30.0%	49	5.9%	

The association between years of experience and patient safety grade is similar to an inverted J-shaped association. Respondents with less than 1 year of experience were less likely to give "Excellent/Very Good" patient safety grade but this increased as years of experience gradually increased. However, after experience exceeded 16 years, the percentage of respondents giving "Excellent/Very Good" patient safety grade started to decrease gradually (See Table 4).

As detailed in El-Jardali et al. (2010), respondents working at accredited hospitals were more likely to give their units an "Excellent/Very Good" patient safety grade than respondents working at non-accredited hospitals (74.2% vs. 68.8%) (See Table 4).

#### Number of Events Reported

Respondents working in administrative units were most likely to report more than 5 events over the past year (12.9%) while respondents working in surgical units were the least likely to report more than 5 events over the past year (5.7%) (See Table 4). Quality and safety officers had the highest percent reporting of more than 5 events (29.6%) followed by pharmacists (26.2%). It is worth noting that 57.1% of physicians and 57.2% of nurses reported no events over the past year (See Table 4).

Respondents with less than 1 year of experience were the largest group of respondents to report no events over the past 12 months (68.7%). This percentage dropped as years of experience increased and then rose again after experience exceeded 10 years (See Table 4).

Interaction with patients was significantly associated with number of events reported where respondents having no direct contact with patients were more likely to report more than 5 events over the past 12 months (8.1%).

Respondents working at accredited hospitals were more likely to report more than 5 events (7.7%) over the past 12 months as compared to respondents working at non-accredited hospitals (5.9%) (See Table 4).

### Generalized Estimating Equations for the Patient Safety Composite Scores and Respondent and Hospital Characteristics against the Patient Safety Grade and the Number of Events Reported (See Table 5)

**Table 5:** Generalized Estimating Equations

		**Patient Safety Grade**		**Number of Events Reported**	
		**OR (95% CI)**	**P-value**	**OR (95% CI)**	**P-value**
		
Composite	Supervisor/manager expectations and actions promoting safety	1.23 (1.03 - 1.47)	***0.024***	0.93 (0.83 - 1.03)	0.152
	Organizational Learning-Continuous Improvement	1.30 (1.03 - 1.65)	***0.029***	1.11 (0.94 - 1.32)	0.227
	Teamwork Within Hospital Units	1.64 (1.40 - 1.93)	***<0.001***	0.97 (0.85 - 1.12)	0.708
	Communication Openness	1.09 (0.94 - 1.26)	0.256	1.03 (0.92 - 1.15)	0.579
	Feedback and Communication About Errors	1.54 (1.33 - 1.78)	***<0.001***	1.17 (1.03 - 1.32)	***0.013***
	Non-punitive Response to Error	1.02 (0.91 - 1.15)	0.768	1.10 (0.99 - 1.22)	0.066
	Staffing	1.31 (1.16 - 1.48)	***<0.001***	1.05 (0.93 - 1.19)	0.380
	Hospital Management Support for Patient Safety	1.85 (1.53 - 2.28)	***<0.001***	1.02 (0.90 - 1.16)	0.703
	Hospital Handoffs and Transitions	1.36 (1.15 - 1.61)	***<0.001***	0.79 (0.68 - 0.91)	***0.003***
	Teamwork Across Hospital Units	1.14 (0.92 - 1.40)	0.226	0.94 (0.82 - 1.08)	0.409
Position	Physician	0.79 (0.50 - 1.23)	0.292	1.30 (0.83 - 2.03)	0.258
	Pharmacist	1.07 (0.26 - 4.33)	0.925	2.14 (0.77 - 5.97)	0.146
	Other health professions	0.73 (0.67 - 1.49)	0.389	1.22 (0.60 - 2.46)	0.579
	Unit assistant/Clerk/Secretary/Technician	1.79 (1.17 - 2.74)	***0.008***	0.78 (0.55 - 1.13)	0.190
	Administration	0.39 (0.27 - 0.57)	***<0.001***	3.57 (1.99 - 6.40)	***<0.001***
	Quality and Safety	0.93 (0.39 - 2.58)	0.887	3.14 (1.24 - 7.91)	***0.015***
	Other	1.66 (1.12 - 2.48)	***0.012***	1.28 (0.95 - 1.72)	0.104
	Nurse	1		1	
Accredited	Yes	1.13 (0.69 - 1.46)	0.365	1.23 (0.88 - 1.73)	0.232
	No	1		1	
Hospital Size	Small	0.83 (0.88 - 1.14)	0.246	1.29 (0.67 - 2.46)	0.450
	Medium	1.03 (0.70 - 1.42)	0.859	1.22 (0.64 - 2.32)	0.542
	Large	1		1	
N		3653		3207	

#### Patient Safety Grade

Results showed that a one unit increase in the composite score for supervisor/manager expectations and actions to promote patient safety increased the odds of reporting a better patient safety grade by 1.23 (95%CI = 1.03-1.47; P-Value = 0.024). Similarly, the odds of reporting a higher patient safety grade increased by 1.30 (95% CI = 1.03-1.65; P-Value = 0.029) for a one unit increase in the composite score for organizational learning and continuous improvement. Moreover, a one unit increase in the composite score on teamwork within hospital units increased the odds of reporting a higher patient safety grade by 1.64 (95%CI = 1.40-1.93; P-Value < 0.001). The odds of reporting a higher patient safety grade increased by 1.54 (95% CI = 1.33-1.78; P-Value < 0.001) for a one unit increase in the composite score for feedback and communication about errors. The odds of reporting a higher patient safety grade increased by 1.31 (95%CI = 1.16-1.48; P-Value < 0.001) for a one unit increase in the composite score for staffing. A one unit increase in the composites score for hospital management support for patient safety and hospital handoffs and transitions increased the odds of reporting a higher patient safety grade by 1.85 (95% CI = 1.53-2.28; P-Value < 0.001) and 1.36 (95%CI = 1.15-1. 61; P-Value < 0.001) respectively (See Table 5).

Respondents who held positions as unit assistants and clerks had 1.79 higher odds (95%CI = 1.16-2.74; P-Value = 0.008) of reporting a higher patient safety grade as compared to the nurses while those working in the administration had 0.39 lower odds (95%CI = 0.27-0.57; P-Value < 0.001) of reporting a higher patient safety grade. Respondents working in "other" hospital departments had 1.66 higher odds (95% CI = 1.12-2.48, P-Value = 0.012) of reporting a higher patient safety grade.

#### Number of Events Reported

A one unit increase in the composite score for feedback and communication about errors increased the odds of reporting a higher number of events by 1.17 (95%CI = 1.03-1.32; P-Value = 0.013). Similarly, the odds of reporting a higher number of events decreased by 0.79 (95% CI = 0.68-0.91; P-Value = 0.003) for a one unit increase in the composite score for hospital handoffs and transition (See Table 5).

Respondents who reported working in administration had 3.57 higher odds (95%CI = 1.99-6.40; P-Value < 0.001) of reporting a higher number of events compared to nurses. Moreover, respondents working in Quality and Safety had 3.14 higher odds (95%CI = 1.24-7.91) of reporting a higher number of events compared to nurses (See Table 5).

### Linear Mixed Regression for the Patient Safety Composite Scores and Respondent and Hospital Characteristics against the Frequency of Events Reported and the Overall Perception of Safety (See Table 6)

#### Frequency of Events Reported

Mixed linear regression analysis showed that a one unit increase in the score on organizational learning and continuous improvement increased the frequency of events reported by 0.159 (P-Value < 0.001). An increase of 0.044 (P-Value = 0.05) in the composite measuring communication and openness was observed for one unit increase in frequency of events reported. A one unit increase in the score on feedback and communication about errors increased the frequency of events reported by 0.366 (P-Value < 0.001). An increase of 0.040 (P-Value = 0.06) in the frequency of events reported was observed for a one unit increase in the score on non-punitive response to errors. Furthermore, an increase of 0.050 (P-Value = 0.05) in the frequency of events reported was observed for a one unit increase in the score on hospital management support for patient safety. A one unit increase in the score on teamwork across hospital units was found to increase the frequency of events reported by 0.100 (P-Value = 0.001) (See Table 6).

**Table 6:** Linear Mixed Model Regression

		**Frequency of Events Reported**		**Perception of Patient Safety**	
		**Beta (Standard Error)**	**P-value**	**Beta (Standard Error)**	**P-value**
		
Composite	Supervisor/manager expectations and actions promoting safety	-0.019 (0.029)	0.52	0.094 (0.016)	***<0.001***
	Organizational Learning-Continuous Improvement	0.159 (0.029)	***<0.001***	0.129 (0.016)	***<0.001***
	Teamwork Within Hospital Units	0.000042 (0.028)	1.00	0.111 (0.016)	***<0.001***
	Communication Openness	0.044 (0.023)	***0.05***	0.039 (0.013)	***<0.001***
	Feedback and Communication About Errors	0.366 (0.023)	***<0.001***	0.021 (0.013)	0.10
	Non-punitive Response to Error	0.040 (0.021)	***0.06***	0.031 (0.012)	***0.01***
	Staffing	-0.017 (0.023)	0.46	0.120 (0.013)	***<0.001***
	Hospital Management Support for Patient Safety	0.050 (0.026)	***0.05***	0.099 (0.015)	***<0.001***
	Hospital Handoffs and Transitions	0.028 (0.025)	0.26	0.163 (0.014)	***<0.001***
	Teamwork Across Hospital Units	0.100 (0.029)	***0.001***	-0.045 (0.017)	***<0.001***
Position	Physician	-0.042 (0.076)	0.58	-0.075 (0.046)	0.10
	Pharmacist	-0.214 (0.195)	0.27	-0.079 (0.118)	0.50
	Other health professions	0.079 (0.129)	0.54	0.073 (0.078)	0.35
	Unit assistant/Clerk/Secretary/Technician	0.016 (0.053)	0.76	0.054 (0.031)	0.08
	Administration	-0.012 (0.108)	0.91	-0.035 (0.062)	0.57
	Quality and Safety	-0.346 (0.153)	***0.02***	0.045 (0.094)	0.63
	Other	-0.001 (0.061)	0.98	0.041 (0.034)	0.22
	Nurse	0		0	
Accredited	Yes	0.010 (0.064)	0.87	0.113 (0.043)	***0.01***
	No	0		0	
Hospital Size	Small	0.069 (0.082)	0.41	-0.035 (0.058)	0.54
	Medium	0.026 (0.086)	0.76	0.0001 (0.061)	1.00
	Large	0		0	
N		4046		3971	

Respondents working in the quality and safety departments had lower frequency of events reported (beta = -0.364, P-Value = 0.020) as compared to nurses (See Table 6).

#### Overall Perception of Safety

Perception of patient safety improved by 0.094 (P-Value < 0.001) for a one unit increase in the score on supervisor/manager expectations and actions promoting safety, by 0.129 (P-Value < 0.001) a one unit increase in the score on organizational learning and continuous improvement, and by 0.111 (P-Value < 0.001) for a one unit increase in the score on teamwork within hospital units. Similarly, perception of patient safety also improved by 0.031 for a one unit increase in the score on non-punitive response to error (P-Value = 0.01), 0.120 for staffing (p-Value < 0.001), 0.099 for hospital management support for patient safety (p-Value < 0.001) and 0.163 for hospital handoffs and transitions (p-Value < 0.001). However, perception of patient safety decreased by -0.045 (P-Value < 0.001) for a one unit increase in the score on teamwork across hospital units (See Table 6).

Respondents working as unit assistant/clerk/secretary/technicians had 0.054 (P-Value = 0.08) better perception of patient safety as compared to nurses (See Table 6).

Accredited hospitals were found to have better perception of patient safety as compared to non-accredited hospitals (beta = 0.113, p-value = 0.01) (See Table 6).

## Discussion

The HSOPSC is one of the most common tools being used to assess the culture of safety in hospitals. Studies that utilize this tool usually report the 12 composite scores and the scores on the patient safety grade and the number of events reported. However, exploring the association between the patient safety composite scores and the hospital and respondent characteristics with the patient safety culture outcomes are not common in the literature. To our knowledge, this is one of the few studies that explore such an association. The analysis of results identified that patient safety culture predictors such as event reporting, proper communication, patient safety leadership and management, hospital size, and accreditation status are associated with the patient safety culture outcomes.

### Event Reporting

A safety culture includes three major components a just culture, a reporting culture, and a learning culture [11]. Event reporting, an essential component for achieving a learning culture, can only happen in a non-punitive environment where events can be reported without people being blamed [12]. The non-punitive response to error composite received one of the lowest scores revealing that Lebanese hospital employees are not at ease when it comes to reporting errors.

The majority of physicians and nurses in our sample reported no events over the past 12 months. Training opportunities that empower physicians to improve patient safety are limited thus investing in the training of physicians is important because they can play a major role in improving patient safety initiatives primarily by improving patient safety outcomes and reducing hassles and wasted time [13].

According to Leape et al. (1995), errors will be more frequent if nurses did not intercept 86% of all potential errors [14]. However, the majority of nurses in our sample reported no events over the past 12 months. Many errors in health care go unreported for many reasons including fear, humiliation, the presence of a punitive response to error, and the fact that reporting will not usually result in actual change [15]. Encouraging health professionals, specifically nurses, to report events in a non-punitive environment is crucial for improving patient safety.

In our study, the fewest number of respondents to report more than 5 events over the past 12 months worked in surgical units. Errors in operating rooms are not uncommon and can sometimes be catastrophic [16]. Creating a patient safety culture in surgical units by improving communication and reporting more events is a high priority for operating room staff and hospitals [16].

Employees who do not deal directly with the patient are more at ease when it comes to reporting errors. As mentioned in Jones et al. (2008), work in laboratory units is considered as more organized than other units since it is controlled by more professional standards and because errors investigated in these units are done as a group [17]. On the contrary, when an error is performed by a nurse, the nurse is investigated as an individual rather a member of a medical team [17].

Work experience at the hospital also had some impact on the frequency and number of events reported. Frequency of events reported was found to increase with increasing years of experience. On the other hand, the score for perception of patient safety decreased as experience in the hospital increased. The perception of safety is defined as the extent to which procedures and systems are good at preventing errors and the lack of patient safety problems. As people become more experienced, they become more aware of the safety practices undertaken in the institutions they work in. When the perception of safety decreases with the increase in the years of experience, it means that the staff members do not agree that the patient safety practices, systems, and procedures in the hospitals act as barriers to errors and problems. A study by Bodur and Feliz (2009) showed that patient safety culture scores decreased as seniority increased [18]. The observation may be the result of an increase in medical errors done by the respondent due to frustration with hospital regulations or increasing staff awareness of safety problems and thus additional reporting.

### Communication

Proper communication within and across healthcare teams is essential to remove any threats to safety of patients. Communication problems have been identified as major contributing factors to adverse events [7]. An analysis of 2,455 sentinel events reported to the Joint Commission on the Accreditation of Healthcare Organizations showed that 70% of the cases were a result of failure in communication [19]. In the absence of proper communication between the different hospital units, patient safety might be jeopardized. Our results revealed that higher scores on teamwork across hospital units increase the frequency of events reported. Moreover, higher scores on hospital handoffs and transitions increased the likelihood of having a better perception of safety among respondents and the likelihood of respondents to report a higher patient safety grade.

### Patient safety leadership and management

In order for a patient safety program to be successful, strong leadership is needed. Senior leaders are the only ones who are able to create the culture and commitment needed to solve underlying system causes of medical errors and harm to patients. When leadership and management is committed to a culture of safety, the whole organization will follow and thus disclosing adverse events and finding their root causes will become an organizational process. The focus should be in emergency rooms, operating rooms, and intensive care units [20]. Our results showed that more support from hospital management for patient safety increased the frequency of events reported. It also increased the likelihood of having a better overall perception of safety among respondents and the likelihood of respondents to report a higher patient safety grade.

### Staffing

Staffing, a major predictor of patient safety, was one of the composites that received a very low score. Having a strong, capable, and motivated workforce is one of the biggest challenges for hospitals today [21]. Evidence has shown that a strong link exists between the availability of health care providers and population health outcomes [21]. According to Sandars & Cook (2007), major catastrophes have occurred in organizations with insufficient staffing. Medical personnel in under-staffed hospitals are overworked [7]. They also suffer from stress and sleeplessness which might lead to lapses in performance thus leading to errors affecting quality and performance [22]. Our results showed that a more positive score on staffing increased the likelihood of having a more positive perception of safety among respondents and the likelihood of respondents to report a higher patient safety grade.

### Hospital size and accreditation

Hospital size and accreditation status were also factors affecting the culture of safety in hospitals. Small hospitals scored higher than larger hospitals on both the frequency of events reported and on the overall perception of safety. Large hospitals usually face challenges when it comes to implementing quality work especially because of bureaucracy. On the contrary, small hospitals have a more homogenous culture where staff members are more likely to share the same values [23].

Accredited hospitals also scored higher on both frequency of events reported and on the overall perception of safety in comparison to non-accredited hospitals. Respondents working at accredited hospitals had an increased likelihood of having a more positive perception of safety. Since the development of hospital accreditation programs that made accreditation a requirement for financial reimbursement by the MOPH, quality improvement initiatives have gained increased attention in Lebanese hospitals [24]. A study by El-Jardali et al. (2008) showed that Lebanese nurses perceived that accreditation created an improvement in the quality of health care [25].

### Limitations

A methodological limitation pertaining to this study should be acknowledged. In the literature, it is argued that social and behavioral research, particularly those that include self-reports such as surveys, is prone to Common method variance (CMV). CMV is believed to pose a threat to the validity of the data as it may either inflate or deflate the correlations among research variables. Although this issue can be attended to during survey development, evidence in the literature stipulate that although such research methods are more prone to CMV, they should not be considered weak or inappropriate if the researchers follow rigorous research conventions in research design, data collection and analysis [26]. In our study, CMV was avoided by the following [26]:

- Assuring that the composite scores were derived based on a combination of items;

- Counterbalancing the order of the questions;

- Ensuring the confidentiality and anonymity of respondents;

- Using clearly written scale items to make responses less subject to bias; and

- Informing respondents in the consent form that there is no preferred or correct answer, and that their individual responses would not be viewed by their management and survey completion would not affect their status at their hospital so they would be encouraged to honestly assess and respond freely

## Conclusion

Investing in patient safety practices in Lebanese hospitals is crucial. While patient safety is everyone's concern, it is not easy for everyone who works in health care organizations to understand this concept. In Lebanon, most health workers have had different training, and often hold a value system that is specific to their professional group. To be truly effective, patient safety needs to be incorporated into the education of health professionals across the spectrum of health care.

Our results demonstrate that patient safety should be a top strategic priority for the health care organizations and its leaders. There should be blame-free system for identifying threats to patient safety, sharing information and learning from events. In addition, there should be a collaborative environment so that all health workers in the healthcare organization can share and exchange information about patient safety

To facilitate change in cultural behaviors, hospital management should assess and redesign their current patient safety system including governance and reporting structures. In addition, they should provide their health professionals with comprehensive training on patient safety concepts, tools, and implementations.

Our results also show that the move to prioritize patient safety by healthcare systems through accreditation is important. However, there is still a good deal skepticism on the part of health professionals who feel that by sharing highly sensitive information they may still be subject to blame and penalties. Progress in this respect will need far more enlightened policies, where health professionals are actively encouraged to report errors for the purpose of learning and improvement. In Lebanon, it is important to strengthen the new accreditation chapter on patient safety by supporting hospitals in training their staff, especially the less experienced ones, on patient safety competencies and about effective implementation of the new standards. Further research is needed to study the association between patient safety culture and clinical outcomes.

It is hoped that the outcome of this study will help conduct a policy dialogue meeting for policy makers and stakeholders in Lebanon to discuss the findings and make deliberation about potential next steps. Senior policy makers, managers and leaders are the only ones who are able to create the culture and commitment needed to identify and resolve underlying systemic causes related to patient safety.

## List of abbreviations used

IOM: Institute of Medicine; HSOPSC: Hospital Survey on Patient Safety Culture; MOPH: Ministry of Public Health;

## Competing interests

The authors declare that they have no competing interests.

## Authors' contributions

FE was the principal investigator on this study and contributed to the conception, design, as well as analysis and interpretation of results. HD was involved in conceptualizing and implementing the data analysis plan as well as overseeing drafting of research results. DJ made substantial contributions to project management, including data collection and analysis as well as in drafting the manuscript. MJ was in charge of project management including data collection, she also contributed to data analysis and write-up of the final version of the paper. NH contributed to data entry and analysis and also made major contributions to the write up of the paper. All authors have read and approved the final version of the manuscript.

## Pre-publication history

The pre-publication history for this paper can be accessed here:

http://www.biomedcentral.com/1472-6963/11/45/prepub

## Supplementary Material

Additional file 1 **Description of Patient Safety Culture Composites and Cronbach's Alpha**. File contains a definition of each of the Patient Safety Culture Composites in addition to Cronach's Alpha for each of the composites. Click here for file
