# Supplementary material for: Predictors and outcomes of patient safety culture in hospitals
Source: BMC Health Serv Res. 2011 Feb 24;11:45. doi: 10.1186/1472-6963-11-45 (PMC3053221; doi:10.1186/1472-6963-11-45)
Supplement: Additional file 1 — Description of Patient Safety Culture Composites and Cronbach's Alpha. File contains a definition of each of the Patient Safety Culture Composites in addition to Cronach's Alpha for each of the composites. [file 1472-6963-11-45-S1.DOC]

| **Box 1** | | |
| --- | --- | --- |
| **Patient Safety Culture Composites** | **Cronbach’s Alpha** | **Definition: The extent to which….** |
| Communication openness | 0.460 | Staff freely speak up if they see something that may negatively affect a patient, and feel free to question those with more authority |
| Feedback & communication about error | 0.645 | Staff are informed about errors that happen, given feedback about changes implemented, and discuss ways to prevent errors |
| Frequency of events reported | 0.809 | Mistakes of the following types are reported: 1) mistakes caught and corrected before affecting the patient, 2) mistakes with no potential to harm the patient, and 3) mistakes that could harm the patient, but do not |
| Handoffs & transitions | 0.739 | Important patient care information is transferred across hospital units and during shift changes |
| Management support for patient safety | 0.631 | Hospital management provides a work climate that promotes patient safety and shows that patient safety is a top priority |
| Non-punitive response to error | 0.534 | Staff feel that their mistakes and event reports are not held against them, and that mistakes are not kept in their personnel file |
| Organizational learning – Continuous improvement | 0.499 | There is a learning culture in which mistakes lead to positive changes and changes are evaluated for effectiveness |
| Overall perceptions of patient safety | 0.451 | Procedures and systems are good at preventing errors and there is a lack of patient safety problems |
| Staffing | 0.479 | There are enough staff to handle the workload and work hours are appropriate to provide the best care for patients |
| Supervisor/manager expectations & actions  promoting safety | 0.568 | Supervisors/managers consider staff suggestions for improving patient safety, praise staff for following patient safety procedures, and do not overlook patient safety problems |
| Teamwork across units | 0.684 | Hospital units cooperate and coordinate with one another to provide the best care for patients |
| Teamwork within units | 0.684 | Staff support one another, treat each other with respect, and work together as a team |
| Cronbach’s alpha for this study extracted from reference # 9: El-Jardali F., Jaafar M., Dimassi H., Jamal D., Hamdan R. The Current state of Patient Safety Culture in Lebanese Hospitals: A study at Baseline. International Journal for Quality in Health Care. 2010; pp. 1–10  Definitions extracted from reference: Battles et al.: Results from the 2009 AHRQ Hospital Survey on Patient Safety Culture, International Forum on Quality and Safety in Health Care: Berlin, Germany; 2009. | | |
